# High-Efficiency, Broadband, Near Diffraction-Limited, Dielectric Metalens in Ultraviolet Spectrum

**DOI:** 10.3390/nano10030490

**Published:** 2020-03-09

**Authors:** Saima Kanwal, Jing Wen, Binbin Yu, Dileep Kumar, Xu Chen, Yi Kang, Chunyan Bai, Dawei Zhang

**Affiliations:** 1 Engineering Research Center of Optical Instrument and Systems, Ministry of Education and Shanghai Key Lab of Modern Optical System, University of Shanghai for Science and Technology, No. 516 Jun Gong Road, Shanghai 200093, China; 142499021@st.usst.edu.cn (S.K.); 161390013@st.usst.edu.cn (B.Y.); 191380026@st.usst.edu.cn (X.C.); ky930827@sina.com (Y.K.); baichunyan1984@163.com (C.B.); 2 State Key Laboratory of Industrial Control Technology, College of Control Science and Engineering, Zhejiang University, Hangzhou 310027, China; dk2kes21@gmail.com; 3 Shanghai Institute of Intelligent Science and Technology, Tongji University, Shanghai 200082, China

**Keywords:** UV, metalens, broadband, diffraction-limited, PB phase

## Abstract

Ultraviolet (UV) optical devices have plenteous applications in the fields of nanofabrication, military, medical, sterilization, and others. Traditional optical components utilize gradual phase accumulation phenomena to alter the wave-front of the light, making them bulky, expensive, and inefficient. A dielectric metasurface could provide an auspicious approach to precisely control the amplitude, phase, and polarization of the incident light by abrupt, discrete phase changing with high efficiency due to low absorption losses. Metalenses, being one of the most attainable applications of metasurfaces, can extremely reduce the size and complexity of the optical systems. We present the design of a high-efficiency transmissive UV metalens operating in a broadband range of UV light (250–400 nm) with outstanding focusing characteristics. The polarization conversion efficiency of the nano-rod unit and the focusing efficiency of the metasurface are optimized to be as high as 96% and 77%, respectively. The off-axis focusing characteristics at different incident angles are also investigated. The designed metalens that is composed of silicon nitride nanorods will significantly uphold the advancement of UV photonic devices and can provide opportunities for the miniaturization and integration of the UV nanophotonics and its applications.

## 1. Introduction

Metasurfaces have stirred up a spree of research interest in recent years due to their brilliant performance in the field of electromagnetic wave manipulation [1,2,3,4,5,6,7,8,9]. Metasurfaces are based on some well-designed subwavelength scale arrays of resonators to manipulate the amplitude, phase, propagation direction, and polarization of light to nanoscale resolution at an ease [10,11,12,13,14,15,16,17,18,19], making them an appropriate option for miniaturization and integration of photonic systems. Currently, metasurfaces are being applied to various applications, such as metalenses [20], holograms [21,22], cloaking [23,24], surface plasmon launcher [4], nonlinear devices [3,11,25,26,27,28], bio sensing [26,27], computing [11,28], switching [5,29], and various novel photonic systems and devices [30,31,32,33,34]. Among these, metalenses are a stirring and significant research direction and application of metasurfaces since they not only outperform the optical properties but are also far smaller and ultrathin comparing to the conventional expensive and bulky optical lenses [35]. Furthermore, they provide a doorway to the integration and miniaturization of the optical devices. Transmissive or reflective metalenses can be explored by dielectric or plasmonic metasurfaces. Plasmonic metalenses perform well in reflection mode. However, owing to the higher reflection and absorption losses of the metals, plasmonic metalenses are less efficient in much preferred ‘transmissive mode’ for most of the optical devices and systems. The quest for highly efficient photonic devices has led to the employment of dielectric material as the most promising candidate for the progression of high efficiency transmissive metalenses. Optical losses are minimized to a significant extent by choosing a wide bandgap and high refractive index dielectric material. Thus, dielectric metalenses outperform the plasmonic metalenses in terms of transmission applications [36]. The desired characteristics of dielectric metalenses comprise high diffraction and transmission efficiencies, large numerical aperture (NA), and broadband wavelength operation [37]. 

Previously reported work on metalenses has focused on various wavelengths ranging from ultraviolet to near-infrared [38], but these metalenses defocused at a small range of UV spectrum due to the insufficient phase delay. Phase manipulation can be realized by various methods depending on principles; for instance, Huygens metasurfaces [39,40], surface plasmon wave-guiding [41,42] and dielectric effective refracting [43,44]. For the Pancharatnam–Berry (PB) phase method, the extra local phases of unit cells are controlled by incident angles by element rotation, and they manipulate circularly polarized (CP) light with great ability. 

As mentioned above, earlier reported work on the metalenses in the UV range is lean and they do not operate in a broadband UV spectrum, and hence are defocused on a small UV range. Moreover, the focusing efficiency and the polarization conversion efficiency of the unit cell is not as high as reported in our work [38,45,46]. Our design of a 2-D, dielectric UV metalens is based on silicon nitride Si_3_N_4_ metasurface, exhibiting high efficiency with a full phase delay of 2π for the broadband UV range (250–400 nm) using PB phase. Our designed metalens has polarization conversion efficiency as high as 96%, thus enabling ultra-high focusing efficiency to 77%. Furthermore, the calculated full width at half maximum (FWHMs) of the focal spot is also diffraction limited. We envision that the designed metalens will open up a new doorway towards the development of miniaturization and integration of the UV photonic devices and its applications.

## 2. Materials and Methods 

**Figure 1:**
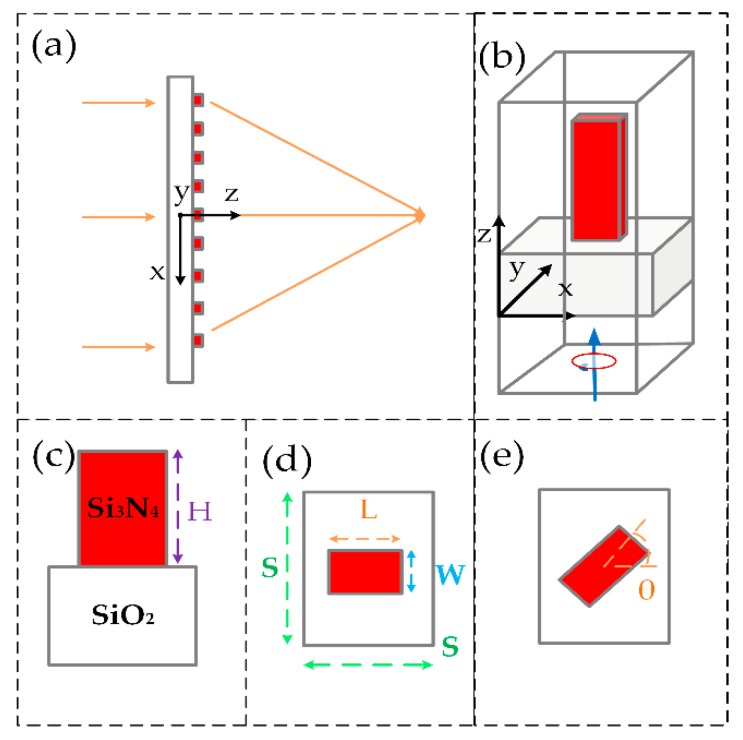
Design of the metalens. (**a**) Schematic of the metalens and its building block, the ${\mathrm{Si}}_{3}N_{4}$ nanorod. (**b**) Si_3_N_4_ nanorod on SiO_2_ substrate. (**c**) and (**d**) side and top views of the unit cell showing the height, width, and length of the nanorod having unit cell dimensions S × S. (**e**) By the rotation of the nanorod, the required phase is imparted by an angle of θ following the geometric Panchratnam–Berry phase.

The capability to realize high efficiency dielectric metalens in the targeted UV region is critically based on the optical properties of the material, as indicated by the complex refractive index, *ñ* = *n* + *ik.* The adopted material should have a high refractive index (*n* > 2) with relatively negligible absorption loss (*k* ≈ 0) at the operating UV regime. Despite a negligible absorption being crucial for high transmission efficiency, a higher refractive index assures strong confinement of the UV light that ultimately provide full (0 to 2π) phase control [47]. The bandgap of traditional dielectric materials is narrow, causing high absorption losses in UV. Gallium nitride and titanium oxide have relatively large bandgaps but they are not appropriate to use in the targeted UV spectrum due to their higher absorption losses [46]. Si_3_N_4_ has been chosen as the dielectric material for its high refractive index of about 2.3, an ultra-wide bandgap of about 5.1 eV, and its transparency window (*k* ≈ 0) for the UV spectrum of 250–400 nm [48]. Phase manipulation can be achieved through various methods relying on principles such as surface plasmon wave-guiding [41,42], dielectric effective refracting [43,44], and metamaterial Huygens surfaces [39,40]. However, the PB phase method has gained significant attention for being wavelength-independent [49,50]. In this approach, all the building blocks of the metasurface have identical size and uniformly transmitted amplitude. When the circularly polarized light is converted into inverse circular polarized light, the transmitted inversed polarized light will have a geometric phase shift that is double of the rotating angle of the nanorod. The metalens design presented here is based on high aspect ratio Si_3_N_4_ nanorods as shown in Figure 1.

The incident circularly polarized light can be partly converted into inversed circularly polarized light that has the geometric phase according to the PB phase method. In our design, the incident light was left circularly polarized light (LCP) and the transmitted light was right circularly polarized light (RCP). In case of incident LCP light, for a nanorod with rotating angle θ the generated phase shift φ will be φ = 2θ. All the simulations were performed using the commercial finite-difference-time-domain (FDTD) method implemented by commercial software ‘FDTD Solutions’(produced by Lumerical Solutions Co. Ltd., Vancouver, BC, Canada) [51]. For the polarization conversion efficiency calculation, periodic boundary conditions were applied to both x and y-direction and perfectly matched layer (PML) is applied to the z-direction. The simulation area was discretized by a 3-D grid mesh using a step size of 0.25 nm in x, y, and z-directions.

**Figure 2:**
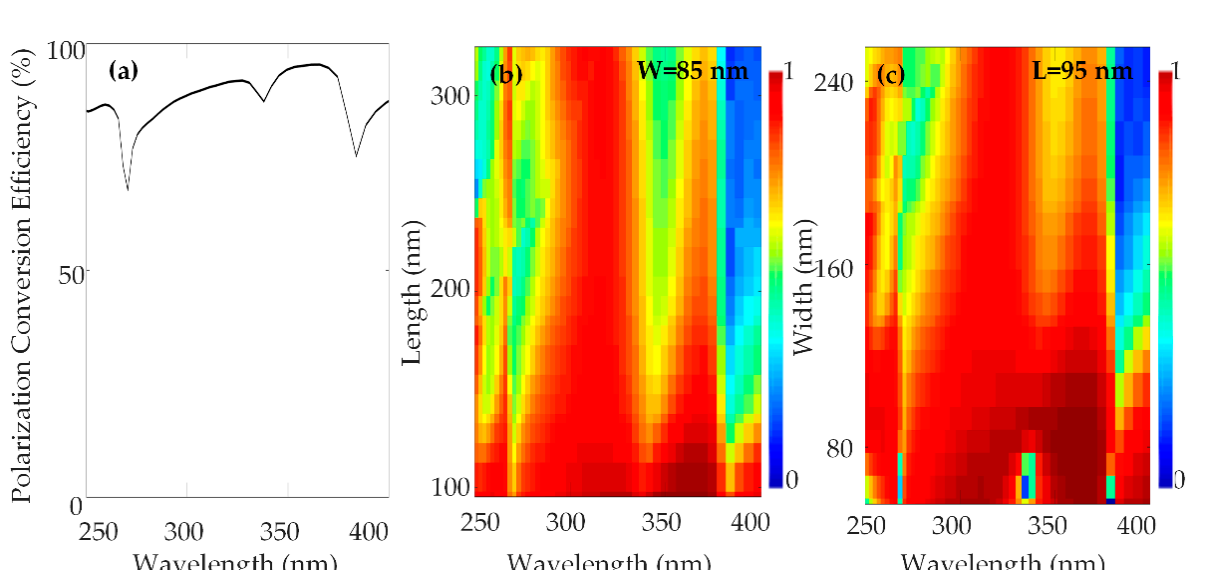
(**a**) Simulated polarization conversion efficiency as a function of wavelength for which periodic boundary conditions were applied in x and y directions and perfectly matched layer boundary conditions were applied in the z-direction. (**b**) Polarization conversion efficiency as a function of wavelength and length of the nanorod at width, W = 85 nm (**c**) Polarization conversion efficiency as a function of the wavelength and width of the nanorod at length, L = 95 nm.

Polarization conversion efficiency can be defined as the fraction of the optical power of the incident circular polarized light which is converted to the optical power of the transmitted inversed circular polarized light [46,51]. By carefully optimizing the unit cell parameters such as height, width, length and period of the nanorod maximum polarization conversion efficiency can be attained. The conversion efficiency of the Si_3_N_4_ nanorod in the wavelength range of 250–400 nm is shown in Figure 2.

The polarization conversion efficiency is as high as 96%. The optimized structure parameters for the unit cell are height H = 210 nm, width W = 85 nm, length L = 95 nm, and period S × S = 240 nm. 

## 3. Design of the Metalens, Results, and Discussion

PB phase method was implemented for designing the broadband UV metalens for light convergence at a broadband range of UV light (i.e., 250–400 nm). The incident light with the wavelength λ is focused to a spot by phase control. For a traditional spherical lens, the difference of refractive index between the different media generates the phase shift. As the light propagates through the surface of metalens to the focal point, different positions in the metalens lead to different optical path difference which has a phase shift relative to the center of the metalens (i.e., [51,52,53]).

(1) $$\varphi (x,y)=\frac{2\pi }{\lambda }(f-\sqrt{f^{2}+x^{2}+y^{2}})$$

where, *x* and *y* are the coordinates of the nanorod in the metalens, *f* is the focal length and λ is the wavelength. As the wavelength and the focal length have been determined, the phase φ*(x,y)* for each nanorod can be calculated. This phase profile is imparted by the rotation of each nanorod at a given coordinate (*x,y)* by an angle θ(*x,y*). According to the Pancharatnam–Berry phase method, the phase shift generated by the rotation of the nanorod at the (*x,y*) position of the metalens has a relationship as [51,52,53]

(2) φ(x,y)=2θ(x,y)

where, θ(*x,y*) is the rotating angle of the nanorod in the position (*x,y*), hence each nanorod in the metalens is rotated by an angle of [51,52,53]

(3) $$\theta (x,y)=\frac{\pi }{\lambda }(f-\sqrt{f^{2}+x^{2}+y^{2}})$$

**Figure 3:**
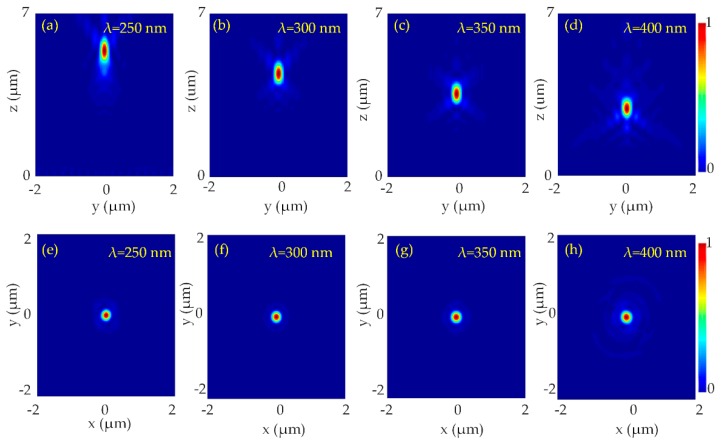
Focusing characteristics of UV metalense at incident UV light 250–400 nm. Normalized intensity distribution at y-z plane at (**a**) λ = 250 nm, (**b**) λ = 300 nm, (**c**) λ = 350 nm and (**d**) λ = 400 nm. Normalized intensity distribution of the metalens at x-y plane at x = y = 0. (**e**) λ = 250 nm along x-axis at z = 5.3 µm, (**f**) λ = 300 nm, along x-axis at z = 4.4 µm (**g**) λ = 350 nm, along x-axis at z = 3.5 µm and (**h**) λ = 400 nm, along x-axis at z = 2.9 µm. The metalens has a diameter of 10 µm, the numerical aperture (NA) of the metalens at designed wavelength λ_d_ = 300 nm is 0.75.

According to the above equation, the rotation angle of all the nanorods is determined at each position. It should be noted that the rotation angle can be an arbitrary value from 0 to π, so the optimized nanorod structure could be able to alter the phase profile of transmitted light at will. Therefore, the transmitted PB phase can achieve full 0 to 2π phase range. The optimized structure parameters for the designed metalens in the wavelength range 250–400 nm were chosen as height H = 340 nm, width W = 85 nm, length L = 150 nm, and period S × S = 240 nm. The diameter of the metalens was 10 µm. The NA of the metalens at the design wavelength λ_d_ = 300 nm was calculated to be 0.75. While focusing, efficiency of the metalens was as high as 77%. Figure 3 presents the focusing characteristics of the designed metalens.

**Figure 4:**
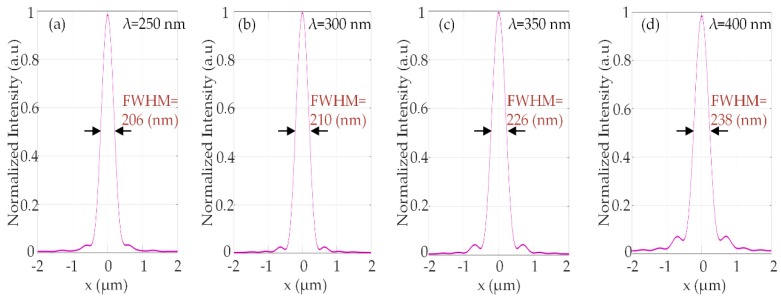
Normalized intensity profiles of the focus spot along x-direction at respective wavelengths (**a**) y = 0, λ = 250 nm, z = 5.3 µm, (**b**) y = 0, λ = 300 nm, z = 4.4 µm, (**c**) y = 0, λ = 350 nm, z = 3.5 µm and (**d**) y = 0, λ = 400 nm, z = 2.9 µm.

The normalized intensity distributions of the transmitted light beam in the y-z section of the metalens are shown in Figure 3a–d and the intensity profiles of the transmitted light beam in the x-y section of the metalens are shown in Figure 3e–h. The intensity distribution of the focal spot exhibits a strongly focused, bright, and symmetric spot at the center of the focal plane at respective wavelengths (250 nm, 300 nm, 350 nm, and 400 nm). The corresponding normalized intensity profiles along the x-axis show a sharp peak at the center of the plane as shown in Figure 4. 

**Figure 5:**
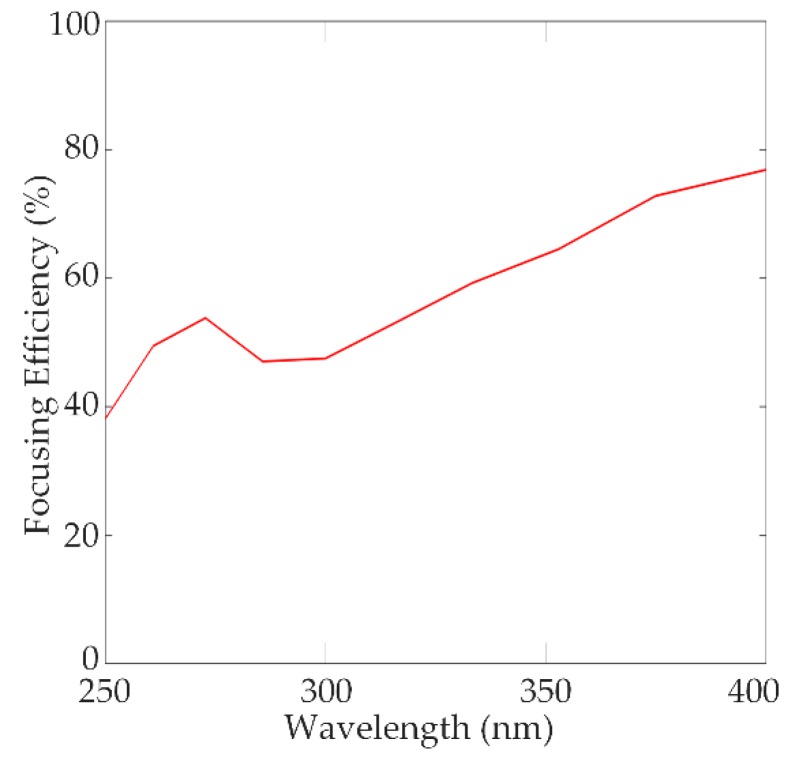
Focusing efficiency of the metalens designed for the broadband UV spectrum 250–400 nm.

The focal length of the metalens was measured at representative wavelengths (250 nm, 300 nm, 350 nm, and 400 nm). The calculated full width at half maximum (FWHM) of the focal spots at the respective wavelengths was 206 nm, 210 nm, 226 nm, and 238 nm; all the values were diffraction-limited (i.e., $\frac{\lambda_{d}}{2NA}$). The normalized intensity profiles of the focal spot along x-direction (at z = *f,* and y = 0) are shown in Figure 4. The focusing efficiency of the UV metalens is measured to be as high as 77%, shown in Figure 5. 

We verified four wavelengths during the optimizing process; the designed metalens worked quite well over the broadband range of 250–400 nm.

**Figure 6:**
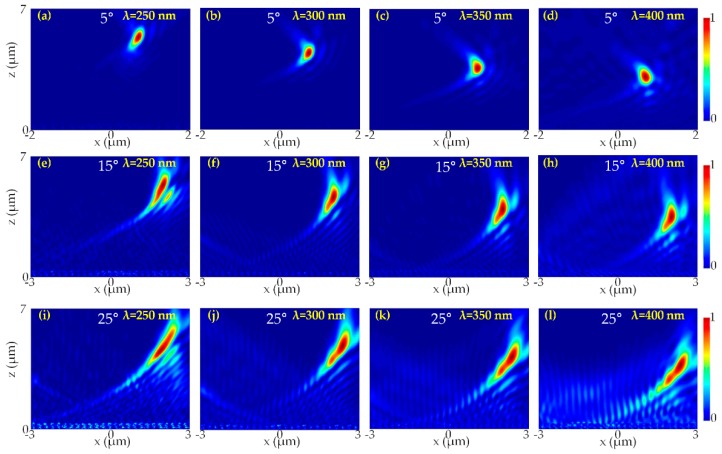
Focusing characteristics of UV metalens at 250–400 nm at different incident angles. Normalized intensity distribution at x-z plane at an incident angle of 5° (**a**) λ = 250 nm, (**b**) λ = 300 nm, (**c**) λ = 350 nm and (**d**) λ = 400 nm. Normalized intensity distribution of the metalens at x-z plane at an incident angle of 15° (**e**) λ = 250 nm, (**f**) λ = 300 nm (**g**) λ = 350 nm and (**h**) λ = 400 nm. Normalized intensity distribution at x-z plane at an incident angle of 25° (**i**) λ = 250 nm (**j**) λ = 300 nm (**k**) λ = 350 nm and (**l**) λ = 400 nm

We also examined the focusing characteristics of the designed metalens at different incident angles (i.e., 5°, 15°, and 25°). It can be seen by the simulation results presented in Figure 6 that the transmitted light beam was clear. Figure 6 shows the focusing characteristics and corresponding normalized intensity distribution of the focal spot at different incident angles at the respective wavelengths.

## 4. Conclusions

In summary, we report the design of a dielectric, high efficiency, diffraction-limited UV metalens based on silicon nitride metasurface functioning in the broadband spectrum of ultraviolet light (i.e., from 250–400 nm). PB phase was implemented to the unit cell to realize the required phase distribution of the metalens. The simulated conversion efficiency was 96% while the focusing efficiency of the metalens was as high as 77%. We also investigated the focusing characteristics of the metalens at different incident angles (i.e., 5°, 15°, and 25°), which showed a clear focus spot. The designed UV metalens can have a great promising perception for a diverse range of applications in lithography, UV laser, UV directional light, image sensors, sterilization, communication, and so on. This research on high efficiency broadband UV metalenses can pave the way towards the advancement of miniaturized and integrated UV nanophotonics. Techniques like multiple couple resonances, tailoring the phase profiles at numerous distinct UV wavelengths, engineering the dispersion, or stacking metasurfaces could be adopted in the future for designing an achromatic UV metalens.
